# Neural correlates of social exclusion and overinclusion in patients with borderline personality disorder: an fMRI study

**DOI:** 10.1186/s40479-023-00240-1

**Published:** 2023-12-01

**Authors:** Adéla Látalová, Monika Radimecká, Martin Lamoš, Martin Jáni, Alena Damborská, Pavel Theiner, Eliška Bartečková, Patrik Bartys, Helena Vlčková, Katarína Školiaková, Tomáš Kašpárek, Pavla Linhartová

**Affiliations:** 1 Department of Psychiatry, University Hospital Brno and Faculty of Medicine, Masaryk University, Brno, Czech Republic; 2 Brain and Mind Research Program, Central European Institute of Technology, Masaryk University, Brno, Czech Republic

**Keywords:** Borderline personality disorder, Rejection sensitivity, Social exclusion, Social overinclusion, Cyberball paradigm, fMRI

## Abstract

**Background:**

Interpersonal difficulties of patients with borderline personality disorder (BPD) are closely related to rejection sensitivity. The aim of the present study was to gain further insight into the experience and cerebral processing of social interactions in patients with BPD by using fMRI during experimentally induced experiences of social exclusion, inclusion, and overinclusion.

**Methods:**

The study involved 30 participants diagnosed with BPD (29 female and 1 male; age: M = 24.22, SD = 5.22) and 30 healthy controls (29 female and 1 male; age: M = 24.66, SD = 5.28) with no current or lifetime psychiatric diagnoses. In the fMRI session, all participants were asked to complete a Cyberball task that consisted of an alternating sequence of inclusion, exclusion, and overinclusion conditions.

**Results:**

Compared to healthy controls, participants with BPD reported higher levels of inner tension and more unpleasant emotions across all experimental conditions. At the neural level, the participants with BPD showed lower recruitment of the left hippocampus in response to social exclusion (relative to the inclusion condition) than the healthy controls did. Lower recruitment of the left hippocampus in this contrast was associated with childhood maltreatment in patients with BPD. However, this difference was no longer significant when we added the covariate of hippocampal volume to the analysis. During social overinclusion (relative to the inclusion condition), we observed no significant differences in a group comparison of neural activation.

**Conclusions:**

The results of our study suggest that patients with BPD experience more discomfort than do healthy controls during social interactions. Compared to healthy participants, patients with BPD reported more inner tension and unpleasant emotions, irrespective of the extent to which others included them in social interactions. At a neural level, the participants with BPD showed a lower recruitment of the left hippocampus in response to social exclusion than the healthy controls did. The reduced activation of this neural structure could be related to a history of childhood maltreatment and smaller hippocampal volume in patients with BPD.

**Supplementary Information:**

The online version contains supplementary material available at 10.1186/s40479-023-00240-1.

## Background

Humans are motivated to engage in social interactions and develop and maintain social connections. A need to belong is fundamental; if this need is not met, it has a negative impact on well-being and mental health [1]. Social exclusion has been proposed as a psychosocial factor significantly contributing to the development and persistence of several mental disorders [2]. One of them is borderline personality disorder (BPD), characterized in part by a pattern of unstable interpersonal relationships and an intense fear of social exclusion [3].

The interpersonal difficulties of patients with BPD are closely related to the concept of rejection sensitivity. Previous research has shown that patients with BPD approach social situations with expectations of rejection that make them hypervigilant for signs of potential exclusion [4–6]. Once confronted with clues of potential rejection, patients with BPD tend to see those signs as more threatening and they experience more distress and anger than healthy controls do [5, 7]. Patients with BPD also tend to respond to actual or perceived rejection with maladaptive behavior, that may include self-mutilation, withdrawal, or hostile behavior [5, 8, 9]. Consistent with these results, a recent meta-analysis identified a moderate relationship between BPD and rejection sensitivity after correction for publication bias (r = 0.338, *p* < 0.001, number of included studies: 52). Patients with BPD had significantly higher sensitivity to rejection when compared with healthy controls (r = 0.784, *p* < 0.001, number of included studies: 12) as well as when compared with patients with other mental health conditions, including social anxiety disorder and current mood disorder (r = 0.294, *p* < 0.01, number of included studies: 12; [10]). In addition, there is a notable link between BPD, sensitivity to rejection, and maltreatment in childhood. According to the rejection sensitivity model proposed by Feldman and Downey [11], high rejection sensitivity is often associated with early experiences of social rejection by primary caregivers, including neglect or abuse. This may overlap with the invalidating environments often observed in the childhood of patients with BPD [12, 13].

Sensitivity to rejection has been studied with different experimental tasks. One of the most commonly used paradigms is a Cyberball game [14] that makes it possible to evaluate participant responses to social inclusion and exclusion. In this task, participants are asked to play a virtual ball-tossing game with two other players, and they are led to believe that they are interacting with real participants. However, the game is pre-programmed; depending on the condition, the actual participant either receives the same number of ball tosses as the other “players” (inclusion condition) or the participant does not receive the ball again after a few initial throws (exclusion condition).

Previous neuroimaging studies identified several brain regions that were involved in the neural processing of social exclusion in healthy participants. A recent meta-analysis that incorporated 53 Cyberball studies revealed increased activation in the bilateral ventral anterior cingulate cortex (vACC), right posterior insula, right superior frontal gyrus (SFG), left inferior frontal gyrus (IFG), left posterior cingulate cortex (PCC), and left occipital pole [15]. There has been some evidence of recruitment of the anterior insula and the dorsal anterior cingulate cortex (dACC; [16]). However, meta-analyses did not identify the involvement of these two regions in the neural processing of social exclusion [15, 17]. Although the precise mechanisms by which these regions are involved in social exclusion remain unknown, existing evidence suggests that increased activity in the vACC is associated with processing emotions and distress induced by social rejection [16, 18, 19]. Enhanced neural activity in the IFG and SFG is linked to the cognitive regulation of feelings after social exclusion [15, 16, 19]. Together with the PCC, the inferior and superior frontal gyri are thought to be related to mentalizing processes and ﻿autobiographical recollection [15, 20, 21].

Neuroimaging studies focusing on the neural substrates of social exclusion in patients with BPD have reported mixed results. In some Cyberball studies, participants with BPD showed increased activation in the pregenual anterior cingulate cortex (preACC; [22]), dorsomedial prefrontal cortex (dmPFC; [23]) and dorsolateral prefrontal cortex (dlPFC; [24]), premotor cortex (PMC; [23]), and precuneus [22], compared to healthy controls. On the other hand, Fertuck and colleagues [24] did not observe any significant differences between BPD patients and healthy controls in neural responses to exclusion events. However, they identified differences in modulation of this neural response through rejection distress. As the level of context-specific rejection distress increased, the response of the rostromedial prefrontal cortex (rmPFC) to exclusion events decreased in participants with BPD but not in healthy controls. The heterogenous results of neuroimaging studies could be explained by the different designs of the Cyberball task. While a majority of studies employed a block design [22–24], Fertuck and colleagues [25] used an event-related design with parametric modulation of exclusion probability. Although the mechanism by which these regions are involved in the processing of social rejection remains unknown, Rappaport and Barch [26] pointed out that the neural response to social exclusion ﻿in BPD patients was associated mainly with increased activity of regions in the default mode network (DMN) and suggested that the hyperactivation of the DMN could be related to negative self-referential processing following social exclusion.

Moreover, growing evidence shows that in patients with BPD, the activation of regions involved in the neural processing of exclusion is increased even during social inclusion. Compared to healthy controls, patients with BPD showed higher neural activation within the anterior insula, dmPFC, dlPFC, PMC, and the precuneus when socially included (relative to passive watching; [4, 23, 27]). Increased activation in most of these regions was significant even in comparison with patients with nonsuicidal self-injury (NSSI; [4, 23]) and/or major depressive disorder (MDD; [27]). Differences in the cerebral processing of social inclusion are in line with the subjective feelings of rejection, higher levels of negative emotions, and lower sense of belonging that patients with BPD reported during inclusion conditions [5, 22, 24, 28].

Some evidence has suggested that patients with BPD need to be overincluded in social situations to experience a decrease in unpleasant emotions to a level comparable to that of healthy controls. During the overinclusion condition in the Cyberball paradigm, in which most of the ball passes are directed to the participant [14], patients with BPD reported reduced levels of negative emotions (relative to the inclusion condition). However, the feeling of social connection and the satisfaction of social needs did not differ between conditions and were significantly lower when compared with healthy controls [28, 29]. A recent EEG study examined the effects of social overinclusion on the event related potential P2, which has been linked with the processing of rewarding stimuli. In participants with BPD, the transition from inclusion to overinclusion was associated with an increase in the P2 amplitude, although the level of positive emotions did not differ between conditions [30]. Another EEG study focused on overinclusion and the P3 complex that is related to the processing of expectancy violation. Compared to healthy controls, patients with BPD showed enhanced P3 amplitudes in both the inclusion and overinclusion conditions [29]. Taken together, the results of these studies suggest that increases in the frequency of social interactions are processed as rewarding and lead to the reduction of painful emotions in patients with BPD. However, the effect of the overinclusion experience on positive emotions, satisfaction of social needs, and feelings of social connection might be limited. The experience of social overinclusion may be too brief for patients with BPD for it to affect their long-lasting and deep-seated expectations of rejection. As a result, social situations might be more uncomfortable for patients with BPD, regardless of the extent to which others include them in social interactions.

While the neural correlates of social exclusion and inclusion have been examined in several studies, the neural processing of social overinclusion has received little attention and remains underexplored. The aim of the present study was to gain further insight into the experience of social exclusion and overinclusion and its underlying neural processes in patients with BPD. We used the Cyberball paradigm with alternating blocks of three different conditions: social inclusion, social exclusion, and social overinclusion. As pointed out by Bolling et al. [31], the alternating design of the Cyberball task could eliminate potential confounds arising from scanner drift, motion, and changes in participant attention and fatigue over the course of the scanning session. The alternating Cyberball design also corresponds to naturally occurring social interactions, where the level of social engagement varies over time.

We hypothesized that participants with BPD would feel more inner tension and unpleasant emotions than healthy controls during both the exclusion and the inclusion conditions, but not during the overinclusion condition. At a neural level measured by functional magnetic resonance imaging (fMRI), we expected that the participants with BPD would show differential brain activation during the exclusion condition (relative to the inclusion condition) when compared with healthy controls. In line with previous studies using a similar design, we hypothesized that patients with BPD would show enhanced activation in ACC, dmPFC, and dlPFC during the social exclusion. We also expected that participants with BPD would exhibit distinct patterns of brain activation during the overinclusion condition (relative to the inclusion condition) when compared with healthy controls. We hypothesized that BPD patients would show decreased activation in areas associated with processing of social rejection during the overinclusion condition, as opposed to the inclusion condition.

## Methods

### Participants

This study involved 30 participants diagnosed with BPD (29 female and one male; age: M = 24.2, SD = 5.2) and 30 healthy controls (29 female and one male; age: M = 24.7, SD = 5.3) with no current or lifetime psychiatric diagnoses. Participants with BPD were recruited from outpatients receiving treatment at the Department of Psychiatry of the University Hospital Brno. They were included in the study if they met at least five of the nine DSM-V criteria for BPD and had one or more suicidal behavior and/or NSSI episode in the past six months. Healthy controls were recruited through social media advertisements to match for gender, age, and the highest degree of education with the clinical group. Participants with psychotic disorder, autism spectrum disorder, severe neurological disorder, or any contraindication to fMRI scanning were excluded from the study.

In the clinical group, 28 of the 30 participants with BPD met the criteria for other mental disorders. The most prevalent co-occurring disorders were MDD (50%), substance use disorder (43.3%), social anxiety disorder (33.3%), posttraumatic stress disorder (PTSD, 23.3%), and panic disorder (20%). At the time of assessment, the participants with BPD were undergoing treatment with various kinds of antidepressants (66.7%), mood stabilizers (6.7%), benzodiazepines (26.7%), and antipsychotic medication (43.3%). Medication was not interrupted as the treatment of the participants with BPD was ongoing; however, all participants were asked not to take any sedative medication prior to fMRI scanning. Further details on sample characteristics are provided in Table 1.

**Table 1:** Demographic and clinical characteristics of patients with BPD and healthy controls

	BPD-patients		HC		BPD vs. HC		
	*N* = 30 96.7% female		*N* = 30 96.7% female				
	M	SD	M	SD	Independent t-test		
Age	24.2	5.2	24.7	5.3	t(57) = –0.40	*p* = .688	d = –0.11
BSL-23	51.7	14.0	5.7	5.6	t(36.77) = 16.54	*p* = < .001	d = 10.37
MADRS	24.7	7.1	0.8	1.1	t(30.40) = 18.358	*p* = < .001	d = 5.05
BDI	36.0	9.0	5.9	5.0	t(43.28) = 15.74	*p* = < .001	d = 7.31
BAI	25.4	8.1	5.9	4.3	t(43.25) = 11.50	*p* = < .001	d = 6.33
CTQ	58.1	13.4	32.4	5.8	t(37.87) = 9.51	*p* = < .001	d = 10.29
RSQ	15.3	4.9	6.5	2.9	t(57) = 8.33	*p* = < .001	d = 4.01
UPPS-P	132.0	19.7	81.5	14.4	t(57) = 10.98	*p* = < .001	d = 17.41
DERS	61.5	7.3	31.0	6.6	t(57) = 16.66	*p* = < .001	d = 7.02
Education	N		N		Mann–Whitney U test		
Primary	6		4		U = 310	*p* = .025	r = .31
Lower secondary	4		0				
Higher secondary	16		15				
University	4		11				
Comorbidity	N						
MDD	15						
PTSD	7						
Substance abuse	13						
Panic disorder	6						
Social anxiety	10						
OCD	4						

*Note*. *BPD* borderline personality disorder, *HC* healthy control, *N* number of participants, *M* mean, *SD* standard deviation, *BSL-23* Borderline Symptom List, *MADRS* Montgomery-Åsberg Depression Rating Scale, *BDI* Beck Depression Inventory, *BAI* Beck Anxiety Inventory, *CTQ* Childhood Trauma Questionnaire, *RSQ* Rejection Sensitivity Questionnaire, *UPPS-P* Impulsive Behavior Scale, *DERS* Difficulties in Emotion Regulation Scale, *MDD* major depressive disorder, *PTSD* posttraumatic stress disorder, *OCD* obsessive–compulsive disorder

### Procedure

This study was part of a larger project focusing on neural mechanisms of dialectical behavior therapy in patients with BPD. The procedure was conducted in accordance with the Declaration of Helsinki, and it was approved by the Ethics Board of the Faculty of Medicine, Masaryk University, and by the Ethics Board of the University Hospital Brno. All participants agreed with their involvement in the study and provided written informed consent. At the first session, participants were asked to complete a set of self-report questionnaires and they underwent a semi-structured interview with a trained psychiatrist or psychologist working under supervision. The following fMRI session was combined with a simultaneous EEG measurement. During this session, participants completed several tasks, including the Cyberball paradigm. In this article, we present only the part relevant to processing social interactions. Other tasks and EEG data are not reported in this article.

### Psychometric measurements

The diagnosis of BPD was verified by a trained psychiatrist or psychologist working under supervision using the Structured Clinical Interview for DSM-5 Personality Disorders (SCID-5-PD; [32]). Severity of depressive symptoms was assessed using the Montgomery-Åsberg Depression Rating Scale (MADRS; [33]), and information about the quantity and severity of suicidal behavior and NSSI episodes was obtained using a modified version of the semi-structured Suicide Attempt Self-Injury interview (SASII; [34]).

Participants were then asked to complete a set of self-report questionnaires. We assessed severity of BPD symptoms using the Borderline Symptom List (BSL-23; [35]; Czech version: [36]), depressive symptoms using the Beck Depression Inventory (BDI; [37]; Czech version: [38]), anxiety symptoms using the Beck Anxiety Inventory (BAI; [39]; Czech version: [40]), severity of different types of childhood trauma using the Childhood Trauma Questionnaire (CTQ; [41]), rejection sensitivity using the Rejection Sensitivity Questionnaire (RSQ; [42]), tendency to impulsive behavior using the Impulsive Behavior Scale (UPPS-P; [43]; Czech version: [44]) and emotion dysregulation using the Difficulties in Emotion Regulation Scale Short Form (DERS-SF; [45]).

### Experimental task

We used the Cyberball task to examine the processing of social interactions in a laboratory setting [14]. Participants were asked to play a virtual ball-tossing game with two other players who were presented as being in another room. In reality, the game was pre-programmed, and participants played with a computer [14, 46]. Participants were represented by a hand at the bottom of the screen, and they could pass the ball to the other players by pressing a corresponding button. The two co-players were represented by animated figures with photographs and names, added to increase the ecological validity of the paradigm [14, 24].

In this study, we implemented the Cyberball task consisting of three different conditions: social inclusion, social exclusion, and social overinclusion. The task was divided into 15 rounds with 5 rounds per condition; each round lasted around 70 s and consisted of 12 throws. Experimental conditions were presented to participants in a pseudo-random order. In the inclusion condition, participants received the ball randomly in 70% of all throws. During the exclusion condition, participants received the ball only once at the beginning of the round and then they were excluded from the game for the remaining 11 throws. In the overinclusion condition, the computer-controlled players exchanged the ball between themselves only once at the beginning of the round and then they kept passing the ball to the participant for the remaining 11 throws.

After each round, participants were asked to indicate their subjective valence of emotional experience (5 points, ranging from *“very unpleasant”* to *“very pleasant”*) and the level of inner tension (5 points, ranging from *“not at all”* to *“very strong”*) on a visual rating scale.

### Functional and structural MRI acquisition

The acquisition was performed on the Siemens Prisma 3 T MR whole-body scanner with 64-channel head-neck coil. A high-resolution structural T1 image was scanned for each participant. This makes it possible to localize the active brain areas more accurately. Parameters of the MPRAGE sequence were 240 sagittal slices, TR = 2300 ms, TE = 2.34 ms, FOV = 256 mm, flip angle = 8°, slice thickness = 1 mm. Functional blood-oxygen-level dependent (BOLD) MR data were acquired in a single scanning session with a T2*-weighted multiecho multiband echo planar imaging (ME MB EPI) sequence of 1250 scans (60 slices, TR = 1000 ms, TE = 14, 34.63 and 55.26 ms, FOV = 200 mm, flip angle = 50°, slice thickness = 2.5 mm, MB factor = 5). TE values were chosen according to recommendations for ME EPI [47], where the second echo was very close to the typical value of single echo acquisition.

### Data analysis

#### Behavioral and self-reported data

Behavioral and self-reported data analysis was performed in Jamovi software version 2.3 [48]. Differences in self-report questionnaires were analyzed by independent t-tests. Differences in ratings of inner tension and valence of emotional experience reported after each block of the Cyberball game were tested by mixed analysis of variance (ANOVA) with the between-subject factor “group” (BPD and HC) and the within-subject factor “Cyberball condition” (exclusion, inclusion, and overinclusion). Post hoc comparisons using Tukey correction were computed to explore the differences motivating the main effects.

#### fMRI data pre-processing

The data were processed with the SPM12 toolbox [49] running under MATLAB R2021a [50]. At first, the realignment procedure was performed to the middle echo scans (all middle echo scans were aligned to the first middle echo scan). The same translations and rotations were used for other echoes. Physiological noise linked to the ECG and respiration were suppressed by RETROICOR in all scans. Composite scans generated from all three echoes were created using the contrast-to-noise weighted average. In each voxel, temporal SNR (tSNR) values were computed for each echo. The resulting voxel value was given by the weighted average of the three original tSNR-weighted values and echoes [51, 52]. The processed functional scans were then co-registered to anatomical scans and all data were normalized to the Montreal Neurological Institute (MNI) template. As a last step, the spatial smoothing of functional data was calculated by Gaussian filter with full width at half maximum (FWHM) of 5 mm. The data quality was checked for the presence of spatial abnormalities in Mask Explorer [53] and for the presence of excessive movement in the movement_info tool [54]. Movement in the data was controlled by a framewise displacement (FD) measure [55]. All the participants were eligible using the thresholds of 20% of scans exceeding FD = 0.5 mm and 1% of scans exceeding FD = 1.5 mm [51].

#### fMRI data analysis

General linear modelling (GLM) was used on the pre-processed data in SPM12 [49]. In the first step, GLM was performed on the subject level. The design matrix contained five time-courses of stimulation design convolved with canonical hemodynamic function and six confound regressors for movement (translations and rotations from the realignment pre-processing procedure). The stimulation task was modelled as a block design, where five time-courses of stimulation represent exclusion blocks, inclusion blocks, overinclusion blocks, a fixating cross screen preceding each round, and a resting block that comes after each round. First level contrasts correspond to condition blocks. In the next step, group-level GLM analysis was performed. According to our hypotheses, we used a two-sample paired t-test to find the differences between exclusion > inclusion and overinclusion > inclusion conditions in both subject groups. To compare these contrasts between subject groups, a flexible factorial design was used. The factors of subject group, condition, and their interactions were modelled. The interaction group and condition were then contrasted. Group results were evaluated with cluster level inference at p (FWE) < 0.05 (with initial cutoff at *p* < 0.005). The results were visualized using xjView toolbox [56].

#### Correlation analyses

The peak MNI coordinate was selected based on fMRI activations. Data for each subject were extracted as the mean of a 5-mm radius sphere around this coordinate. Pearson correlation coefficients were then calculated between activations in the region of interest and clinical scales or self-reported data. In the case of dichotomous variables, logistic regression was used.

## Results

### Self-reported results

The statistical analysis of self-report measures revealed significant differences between the groups (Table 1). Compared to healthy controls, participants with BPD showed significantly higher severity of BPD symptoms, depression, and anxiety. They also reported significantly higher sensitivity to social rejection, higher emotion dysregulation, and a higher tendency to impulsive behavior.

### Behavioral results

**Fig. 1:**
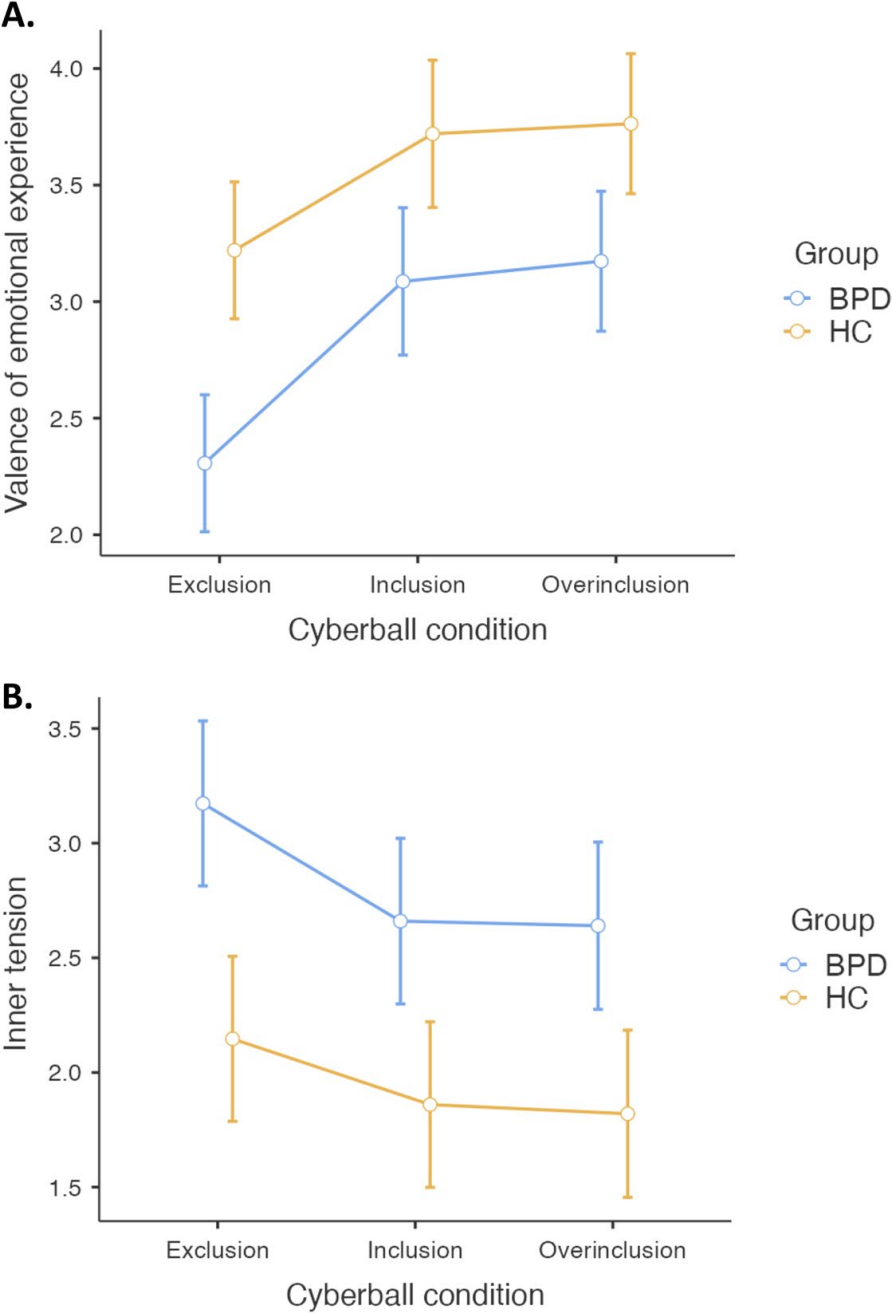
Estimated marginal means plots show average ratings of (**A**) valence of emotional experience and (**B**) inner tension after exclusion, inclusion, and overinclusion conditions reported by participants with BPD (blue) and healthy controls (yellow). *Note*. BPD = borderline personality disorder; HC = healthy control; valence of emotional experience (5 points, ranging from 1 = “very unpleasant” to 5 = “very pleasant”); the level of inner tension (5 points, ranging from 1 = “not at all” to 5 = “very strong”); error bars represent 95% confidence interval

The statistical analysis revealed a significant main effect of group on the valence of emotional experience reported after each block of the Cyberball task, F(1, 58) = 15.5, p =  < 0.001, η^2^ = 0.141. Compared with healthy controls, participants with BPD reported more unpleasant emotions across all experimental conditions, t(58) = -3.94, p_tukey_ =  < 0.001. There was also a significant main effect of condition on the valence of emotional experience, F(1.49, 86.67) = 30.54, *p* =  < 0.001, η^2^ = 0.113. Post hoc comparisons revealed that the exclusion condition was associated with a more unpleasant emotional experience than the inclusion and overinclusion conditions, t(58) = -5.57, p_tukey_ =  < 0.001, respectively t(58) = -6.32, p_tukey_ =  < 0.001. The valence of emotional experience during the inclusion condition did not significantly differ from the overinclusion condition, t(58) = -1.01, p_tukey_ = 0.576. There was no significant interaction effect between group and condition, F(1.49, 86.67) = 1.55, *p* = 0.221. Descriptive statistics and estimated marginal means plots are provided below (Table 2 and Fig. 1.A.).

**Table 2:** Average ratings of valence of emotional experience and inner tension after exclusion, inclusion, and overinclusion conditions in a Cyberball task

Ratings		Cyberball condition		
		Exclusion	Inclusion	Overinclusion
Valence of emotional experience	BPD	M = 2.31	M = 3.09	M = 3.17
		SD = .71	SD = .76	SD = .77
	HC	M = 3.22	M = 3.72	M = 3.76
		SD = .89	SD = .96	SD = .87
Inner tension	BPD	M = 3.17	M = 2.66	M = 2.64
		SD = 1.09	SD = .97	SD = 1.04
	HC	M = 2.15	M = 1.86	M = 1.82
		SD = .86	SD = 1.00	SD = .95

*Note*. *BPD* borderline personality disorder, *HC* healthy control, *M* mean, *SD* standard deviation; valence of emotional experience (5 points, ranging from 1 = “very unpleasant” to 5 = “very pleasant”); the level of inner tension (5 points, ranging from 1 = “not at all” to 5 = “very strong”)

Regarding inner tension (Table 2 and Fig. 1.B.), there was a significant main effect of group, F(1, 58) = 14.0, *p* =  < 0.001, η^2^ = 0.164. Participants with BPD experienced increased inner tension across all experimental conditions when compared with healthy controls, t(58) = 3.75, p_tukey_ =  < 0.001. The statistical analysis revealed a significant main effect of condition, F(1.68, 97.44) = 15.48, *p* =  < 0.001, η^2^ = 0.032. Post hoc comparisons showed that the exclusion condition was associated with a significantly higher level of inner tension than the inclusion and overinclusion conditions, t(58) = 4.353, p_tukey_ =  < 0.001 respectively t(58) = 4.382, p_tukey_ =  < 0.001. The difference between the inclusion and overinclusion conditions was not significant, t(58) = 0.459, p_tukey_ = 0.891. There was no significant interaction effect between group and condition, F(1.68, 97.44) = 1.06, *p* = 0.341.

### fMRI results

#### Exclusion compared to inclusion

The healthy controls showed increased engagement during the exclusion condition as compared to the inclusion condition within two clusters: one cluster comprising the left superior, middle, and inferior temporal gyri (STG, MTG and ITG) and the other cluster encompassing the left middle and inferior frontal gyri (MFG and IFG). The BPD group showed increased engagement within the cluster including the left superior and middle occipital gyri.

**Fig. 2:**
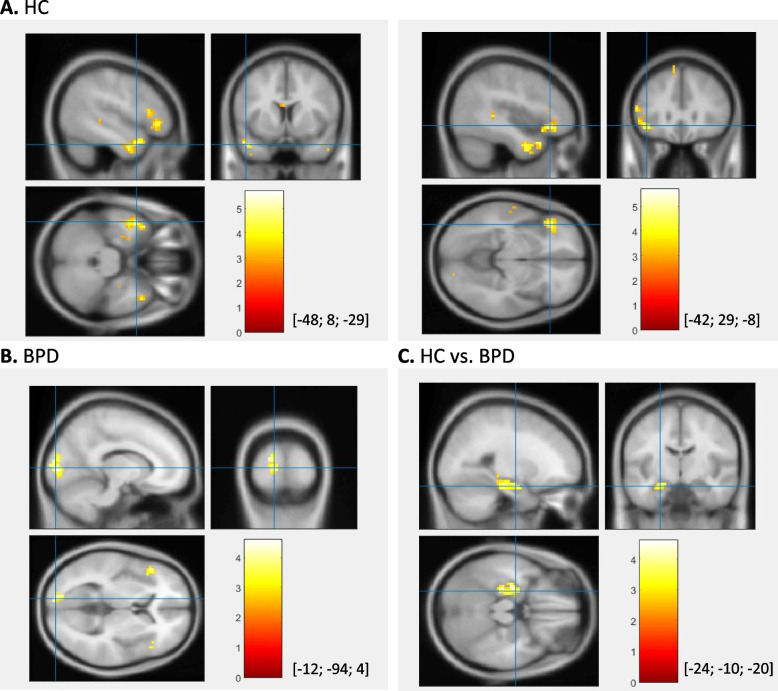
Enhanced neural activation during exclusion compared to inclusion conditions in the Cyberball paradigm. Statistically significant results (p (FWE) < .05 for cluster-level inference, k > 10 voxels) for healthy controls (**A**), patients with BPD (**B**) and comparison between the groups (HC vs. BPD; **C**). Intersection of blue lines indicates the peak of each cluster, MNI-coordinates are provided in the squared brackets. *Note*. BPD = borderline personality disorder; HC = healthy control; MNI = Montreal Neurological Institute (x, y and z coordinates are provided in mm)

When comparing participants with BPD with healthy controls, we did not find any significant increase of neural activation in the participants with BPD; however, we observed enhanced neural reactivity within the left hippocampus in healthy controls during the social exclusion condition relative to the social inclusion condition. Table 3 and Fig. 2 show significant results of the exclusion to inclusion contrast together with the cluster characteristics. Figure 1 in the supplementary material displays the distribution of Cohen’s d for each contrast.

**Table 3:** Enhanced neural activation during exclusion compared to inclusion conditions in the Cyberball paradigm. Statistically significant results (p (FWE) < .05 for cluster-level inference, k > 10 voxels) for HC, patients with BPD and comparison between the groups (HC vs. BPD)

	Anatomic label	Side L/R	MNI			Cluster size	P_FWE-cor_	Cohen’s d
			x	y	z			
HC	Superior temporal gyrus	L	-48	8	-29	107	.015	0.75
	Middle temporal gyrus							
	Inferior temporal gyrus							
	Middle frontal gyrus	L	-42	29	-8	147	.002	0.59
	Inferior frontal gyrus							
BPD	Superior occipital gyrus	L	-12	-94	4	82	.029	0.49
	Middle occipital gyrus							
HC vs. BPD	Hippocampus	L	-24	-10	-20	118	.008	1.05

*Note*. *BPD* borderline personality disorder, *HC* healthy control, *L* left, *R* right, *MNI* Montreal Neurological Institute (x, y and z coordinates are provided in mm); Cluster size = number of voxels

#### Examination of the hippocampal volume influence

Since previous studies found difference in hippocampal volume between patients with BPD and healthy controls [57, 58], we performed several additional analyses to examine whether the difference in neural activation within the left hippocampus was associated with the volume of the structure. First, we calculated the rank sum test that confirmed a significant difference between participants with BPD and healthy controls in the volume of the left hippocampus (rank sum = 730, U = 265, *p* = 0.03). Second, we added the covariate of hippocampal volume to the analysis of task-related activation. The results of this supplementary analysis did not find significant difference between participants with BPD and healthy controls in neural responses to social exclusion relative to social inclusion. A detailed description of the additional analyses can be found in the supplementary material.

#### Overinclusion compared to inclusion

We did not find any significant neural activation when comparing the overinclusion and inclusion conditions in participants with BPD or in healthy controls. Similarly, we did not observe any significant differences in a group comparison of neural activation during the overinclusion condition as compared to the inclusion condition.

### Correlation analyses

We conducted a correlation analysis to examine the association between enhanced neural activation within the left hippocampus and behavioral data and psychometric measurements. In healthy controls, neural activation within the left hippocampus was significantly negatively correlated with BSL-23 behavioral scores (r = -0.440; *p* = 0.015) and BDI scores (r = -0.400; *p* = 0.027). In participants with BPD, we observed a significant negative correlation between neural activation in the left hippocampus and CTQ scores (r = -0.37; *p* = 0.05).

We also performed additional correlation analysis to examine the potential association between hippocampal volume and psychometric measurements. No significant correlations were observed in patients with BPD or healthy controls.

## Discussion

The present study aimed to gain further insight into the experience and the cerebral processing of social interactions in patients with BPD by using fMRI during experimentally induced experiences of social exclusion, inclusion, and overinclusion. Compared to healthy controls, participants with BPD reported higher levels of inner tension and more unpleasant emotions across all experimental conditions. At a neural level, participants with BPD showed lower recruitment of the left hippocampus in response to social exclusion (relative to the inclusion condition) when compared to healthy controls. Lower recruitment of the left hippocampus in this contrast was associated with childhood maltreatment in patients with BPD. However, the difference in the left hippocampal activity between the groups was no longer significant when we added the covariate of hippocampal volume to the analysis. During social overinclusion (relative to the inclusion condition), we did not observe any significant differences in the group comparison of neural activation.

In line with our expectations, during social exclusion and inclusion the participants with BPD experienced higher levels of inner tension and more unpleasant emotions than the healthy controls. These findings are consistent with previous research in which patients with BPD indicated stronger feelings of social rejection, a lower sense of belonging, and more unpleasant emotions during both exclusion and inclusion conditions than did healthy controls [5, 19, 21, 24]. Nevertheless, our initial assumptions regarding social overinclusion were not confirmed. Prior studies of participants with BPD associated overinclusion with a reduction of unpleasant emotions to a level comparable with that of healthy controls. In our study, however, patients with BPD reported increased inner tension and more unpleasant emotions even after experiences of social overinclusion. The mixed results could be partly explained by the different designs of the Cyberball task. Previous studies either applied only the inclusion condition followed by the overinclusion condition [29] or randomly assigned participants into groups with different degrees of social participation [28]. In this study, we used an alternating sequence of the exclusion, inclusion, and overinclusion condition. There is a possibility that the experience of social exclusion may have reduced reward processing and diminished the effect of overinclusion. In fact, similar results were observed in a study with healthy participants who experienced social exclusion, inclusion, and overinclusion during a continuous block of a Cyberball task with an event-related design [19]. Another possible explanation is that the experimentally induced overinclusion may have been too brief for the participants with BPD to affect their deep-seated anticipation of social exclusion. As patients with BPD approach social situations with the expectation of rejection [4, 6] and tend to perceive others as less trustworthy [59, 60], social situations may be more uncomfortable for them, regardless of the extent to which others include them in social interactions.

At a neural level, the healthy participants showed exclusion-evoked neural activation within the cluster comprising the left STG, MTG, and ITG and the cluster including the left MFG and IFG. These areas have previously been reported in other studies using the Cyberball paradigm in nonclinical populations [15, 31]. The activation of these regions during social exclusion could be linked to affective response and cognitive regulation of feelings of rejection [15, 17, 31]. On the other hand, participants with BPD showed increased engagement within the cluster including the left superior and middle occipital gyri during the exclusion condition. Previous studies focusing on social exclusion in BPD patients have not identified enhanced activation in this area. ﻿However, increased neural activation in this region has been observed during social exclusion in healthy participants [15] and has functionally been related to visual processing [61].

Although the participants with BPD experienced more discomfort than the healthy controls across all experimental conditions, these differences were not evident at the neural level. In contrast to our expectations, we did not observe any enhanced activation in the participants with BPD during social exclusion when compared to healthy controls. While some of the prior Cyberball studies identified exclusion-evoked activity within the preACC, dmPFC, dlPFC and precuneus in patients with BPD [22–24], our study did not replicate these findings. However, our results are in line with findings of the recent Cyberball study [25], which used an event-related design with parametric modulation of exclusion probability. Authors of this study did not observe any significant differences between BPD patients and healthy controls in neural responses to exclusion events. However, they identified atypical modulation of the rmPFC response by rejection distress in participants with BPD. Furthermore, our study did not identify any difference between patients with BPD and healthy controls in response to the overinclusion vs. inclusion conditions. Although the level of inner tension and unpleasant emotions was constantly higher in participants with BPD, both groups showed similar neural responses to social overinclusion.

A possible explanation for the lack of more pronounced neural responses to social exclusion and overinclusion could be the alternating design of the Cyberball task. In our study, we used a Cyberball design with alternating blocks of different experimental conditions to eliminate potential confounds arising from scanner drift, motion, and changes in participant engagement over the course of an fMRI session. The meta-analyses comparing traditional and alternating Cyberball designs with healthy participants did not confirm any significant differences between these two approaches [15, 17]. However, some authors have noted the risk of a “spill-over” effect associated with the alternating Cyberball version. They argue that the alternating design could potentially reduce neural differences between the experimental conditions, as the neural response to one condition might affect cerebral processing of other conditions in subsequent blocks [17]. Although the transfer effect has been reported even in studies using a traditional Cyberball design [62], the alternating approach with shorter blocks and repeated switches between different conditions could be more prone to this unintended effect.

Another factor that may have influenced our findings is using social inclusion as the control condition. Although it is a common approach in Cyberball studies [2, 15], some authors question whether the contrast between exclusion and inclusion represents the appropriate way to describe the brain regions that respond specifically to social exclusion [25, 26]. They point out that the interpretation of this contrast is problematic, as it is difficult to separate the specific effects of social exclusion from more general effects associated with processing of social stimuli (e.g. working memory, motor planning or sensory processing; [25]). The same question applies to the contrast between the inclusion and overinclusion conditions. The authors suggest that a better option might be to use a neutral control condition, such as passively watching the game [26], or implementing the event-related design of the Cyberball paradigm [25].

Contrary to our expectations, participants with BPD differed from healthy participants in the activation of the hippocampus during the social exclusion condition. More precisely, compared to healthy controls, participants with BPD showed lower recruitment of the left hippocampus in response to the exclusion vs. inclusion condition. This result was quite surprising, as previous studies of patients with BPD have not reported reduced neural activation in this region. However, there is some evidence for increased hippocampal activation during social exclusion in healthy adult participants [31] and healthy children and adolescents [63]. Since the hippocampus plays an important role in learning, memory encoding and consolidation and emotional processing [64–66], it could be hypothesized that its increased activation reflects healthy participants’ attempts to downregulate painful emotions after social rejection.

Another possible explanation for the lower recruitment of the left hippocampus during social exclusion could be reduced hippocampal volume amongst the participants with BPD. An association between a lower volume of hippocampus and BPD has been reported by several volumetric studies [57, 58]. Existing evidence suggests that the smaller hippocampus size is closely linked to experiences of childhood maltreatment [67–69], which is very common in patients with BPD [70]. In fact, 96.7% of participants with BPD in our study reported some form of childhood maltreatment. Based on these findings, we decided to include hippocampal volume in our additional analysis. In line with previous studies, our results showed that participants with BPD had a reduced volume of the left hippocampus in comparison with the healthy controls. When we added the covariate of hippocampal volume to the analysis of neural response to social exclusion, the difference in neural activation between the groups was no longer significant. These findings suggest that the previously observed lower recruitment of the left hippocampus during social exclusion in patients with BPD could partly reflect a reduced hippocampal volume in this group. Although there is not yet a clear explanation for a smaller hippocampus in patients with BPD, existing evidence suggests that the reduced volume could be associated with long-term differences in the activation of the hippocampal structure in patients with BPD. It also could be linked with impaired feedback inhibition of the hypothalamus–pituitary–adrenal axis and long-term alteration of cortisol levels in participants with BPD [71–73].

Although we did not observe any significant link between hippocampal volume and traumatic experiences during childhood, results of our correlational analysis revealed an association between exclusion-evoked neural activation of the left hippocampus and the childhood maltreatment in patients with BPD. These findings suggest that the initially observed reduced neural activation of the left hippocampus in BPD patients could be partly related to the history of childhood maltreatment. More research is needed to better understand the role of the hippocampus in processing social exclusion in BPD patients. Given the high prevalence of childhood maltreatment in this clinical population, future studies with BPD patients should focus on hippocampal neural activation as well as the volume of this structure.

Several limitations of the present study need to be addressed. As mentioned above, the alternating Cyberball design could potentially lead to reduced neural differences between the conditions, as it may be more prone to a transfer effect than the traditional Cyberball design. Another shortcoming is the absence of a neutral condition in the Cyberball paradigm; this made it difficult to separate task-specific from non-task-specific cognitive processes and prevented us from investigating the neural response to social inclusion. A neutral condition like passively watching the game could represent a better control condition for social exclusion and overinclusion. Nor did our study include a measure of the feeling of social connection and the satisfaction of social needs, which prevented us from capturing potential changes in these variables. There are also a few limitations related to the characteristics of our sample. As our study included mainly female participants (96.7%), our results cannot be easily generalized to male patients with BPD. Consistent with the high prevalence of comorbidity in patients with BPD, 93.3% of the patients in our study met criteria for another mental health condition (most prevalent co-occurring disorders were MDD, substance use disorder, social anxiety, and PTSD). We cannot rule out the potential effects of comorbid disorders on the neural processing of social interactions, as we did not include a clinical control group in this study. In addition, most of the participants with BPD were treated with various types of medication. This could reduce the differences between groups; however, a recent review of the existing literature found little or no effect of pharmacological treatments on the neural processing of emotional tasks in patients with BPD [74].

## Conclusions

To conclude, the results of our study suggest that patients with BPD experience more discomfort than healthy controls during social interactions. Compared to healthy participants, patients with BPD reported more inner tension and unpleasant emotions, irrespective of the extent to which others included them in social interactions. At a neural level, the participants with BPD showed a lower recruitment of the left hippocampus in response to social exclusion than the healthy controls did. The reduced activation of this neural structure could be related to a history of childhood maltreatment and smaller hippocampal volume in patients with BPD.

### Supplementary Information

**Additional file 1:** **Figure 1.** Distribution of Cohen’s d calculated voxel-wise in an exclusion vs. inclusion condition for each contrast: healthy controls (A), patients with BPD (B) and comparison between the groups (HC vs. BPD; C).

## Data Availability

The data that support the findings of this study are not publicly available due to reasons of sensitivity, but they are available from the corresponding author on reasonable request.

## Abbreviations

ANOVA: Analysis of variance
BAI: Beck Anxiety Inventory
BDI: Beck Depression Inventory
BOLD: Blood-oxygen-level dependent
BPD: Borderline personality disorder
BSL-23: Borderline Symptom List
CTQ: Childhood Trauma Questionnaire
dACC: Dorsal anterior cingulate cortex
DERS-SF: Difficulties in Emotion Regulation Scale Short Form
dlPFC: Dorsolateral prefrontal cortex
DMN: Default mode network
dmPFC: Dorsomedial prefrontal cortex
DSM-5: Diagnostic and Statistical Manual of Mental Disorders, Fifth Edition
EEG: Electroencephalogram
FD: Framewise displacement
fMRI: Functional magnetic resonance imaging
FOV: Field-of-view
FWE: Family-wise error
FWHM: Full width at half maximum
GLM: General linear modelling
HC: Healthy control
IFG: Inferior frontal gyrus
ITG: Inferior temporal gyrus
L: Left
M: Mean
MADRS: Montgomery-Åsberg Depression Rating Scale
MDD: Major depressive disorder
ME MB EPI: Multiecho multiband echo planar imaging
MFG: Middle frontal gyrus
MNI: Montreal Neurological Institute
MTG: Middle temporal gyrus
N: Number of participants
NSSI: Nonsuicidal self-injury
OCD: Obsessive–compulsive disorder
PCC: Posterior cingulate cortex
PMC: Premotor cortex
preACC: Pregenual anterior cingulate cortex
PTSD: Posttraumatic stress disorder
R: Right
rmPFC: Rostromedial prefrontal cortex
RSQ: Rejection Sensitivity Questionnaire
SASII: Suicide Attempt Self-Injury Interview
SCID-5-PD: Structured Clinical Interview for DSM-5 Personality Disorders
SD: Standard deviation
SFG: Superior frontal gyrus
STG: Superior temporal gyrus
TE: Time to echo
TR: Repetition time
tSNR: Temporal signal-to-noise ratio
UPPS-P: Impulsive Behavior Scale
vACC: Ventral anterior cingulate cortex
