# Supplementary material for: Neural correlates of social exclusion and overinclusion in patients with borderline personality disorder: an fMRI study
Source: Borderline Personal Disord Emot Dysregul. 2023 Dec 1;10:35. doi: 10.1186/s40479-023-00240-1 (PMC10691118; doi:10.1186/s40479-023-00240-1)
Supplement: Supplementary file 1 — Additional file 1: Figure 1. Distribution of Cohen’s d calculated voxel-wise in an exclusion vs. inclusion condition for each contrast: healthy controls (A), patients with BPD (B) and comparison between the groups (HC vs. BPD; C). [file 40479_2023_240_MOESM1_ESM.docx]

Supplementary material

Methods

Additional analyses

We performed several additional analyses to examine the potential association between task-related activation and hippocampal volume. First, the volumes of the hippocampi were segmented and quantified from T1-weighted brain images utilizing the FreeSurfer software suite (1) version 7.1.0, operating on a Debian 11 GNU/Linux system. Following segmentation, volumes were normalized for individual variability in total intracranial volume using a proportion adjustment method (2). After that, the normality of the hippocampal volume data was tested using the Kolmogorov-Smirnov test and the differences between groups were analyzed using the nonparametric rank sum test. Finally, we added the covariate of the volume of this structure to the analysis of task-related activation.

Results

fMRI results: Exclusion compared to inclusion

| **Figure 1.** Distribution of Cohen’s d calculated voxel-wise in an exclusion vs. inclusion condition for each contrast: healthy controls (A), patients with BPD (B) and comparison between the groups (HC vs. BPD; C). |
| --- |
| **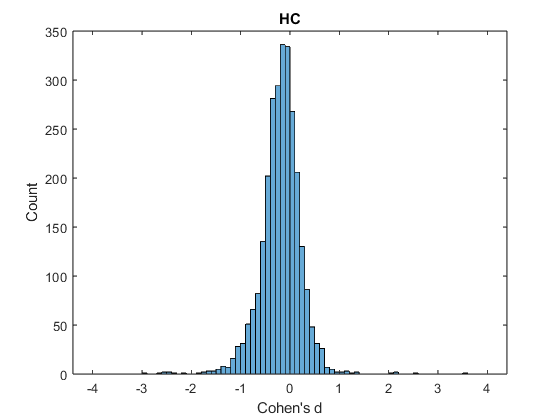A.** |
| **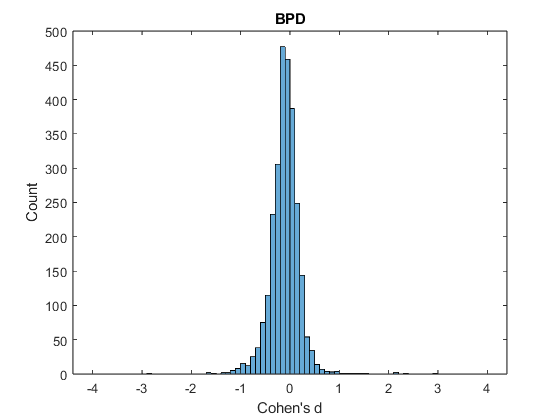B.** |
| **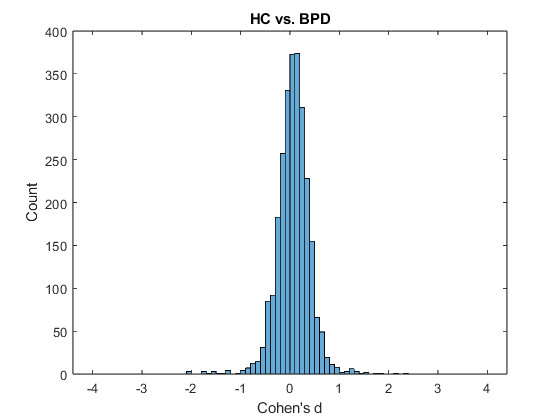C.** |
| *Note*. BPD = borderline personality disorder; HC = healthy control |

References

1. FreeSurfer software suite. http://surfer.nmr.mgh.harvard.edu/. Accessed 20 May 2023
2. Sanchis-Segura C, Ibañez-Gual MV, Aguirre N, Gómez-Cruz ÁJ, Forn C. Effects of different intracranial volume correction methods on univariate sex differences in grey matter volume and multivariate sex prediction. Sci Rep. 2020;10(1):1–15.
